# OpBox: Open Source Tools for Simultaneous EEG and EMG Acquisition from Multiple Subjects

**DOI:** 10.1523/ENEURO.0212-20.2020

**Published:** 2020-10-27

**Authors:** Eyal Y. Kimchi, Brian F. Coughlin, Benjamin E. Shanahan, Giovanni Piantoni, John Pezaris, Sydney S. Cash

**Affiliations:** 1 Department of Neurology, Massachusetts General Hospital, Boston, MA, 02114; 2 Department of Neurosurgery, Massachusetts General Hospital, Boston, MA, 02114; 3 Department of Neurosurgery, Harvard Medical School, Boston, MA, 02115

**Keywords:** amplifier, EEG, electrophysiology, EMG, open source, rodent

## Abstract

*In vivo* electrophysiology experiments require the collection of data from multiple subjects, often for extended periods. Studying multiple subjects for extended periods can be made more efficient through simultaneous recordings, but scaling up recordings to accommodate larger numbers of subjects simultaneously requires coordination and consideration of costs and flexibility. To facilitate this process, we have developed OpBox, an open source set of tools to acquire electroencephalography (EEG) and electromyography (EMG) flexibly from multiple rodent subjects simultaneously. OpBox combines open source hardware and software with off-the-shelf components to create a system that costs less than commercial solutions ($500 per subject), and can be easily deployed for multiple subjects. Coded in MATLAB, OpBox scripts can simultaneously and flexibly collect and display multiple analog and digital data streams, for instance real-time EEG and EMG, event triggers from a behavioral system, and rotary encoder data. OpBox also calculates and displays real-time spectral representations and event-related potentials (ERPs). To verify the performance of our system, we compare our amplifiers with two other commercial amplifiers, a Grass P55 AC preamplifier and an Intan RHD2000-series amplifier. The OpBox amplifier performs comparably to commercial amplifiers for signal-to-noise ratios (SNRs), noise floors, and common mode rejection. We also demonstrate that our acquisition system can reliably record multichannel data from multiple subjects, and has been successfully tested with 12 subjects running simultaneously on a single standard desktop computer. Together, OpBox increases the flexibility and lowers the cost for simultaneous acquisition of electrophysiology data from multiple subjects.

## Visual Abstract

**Figure:**
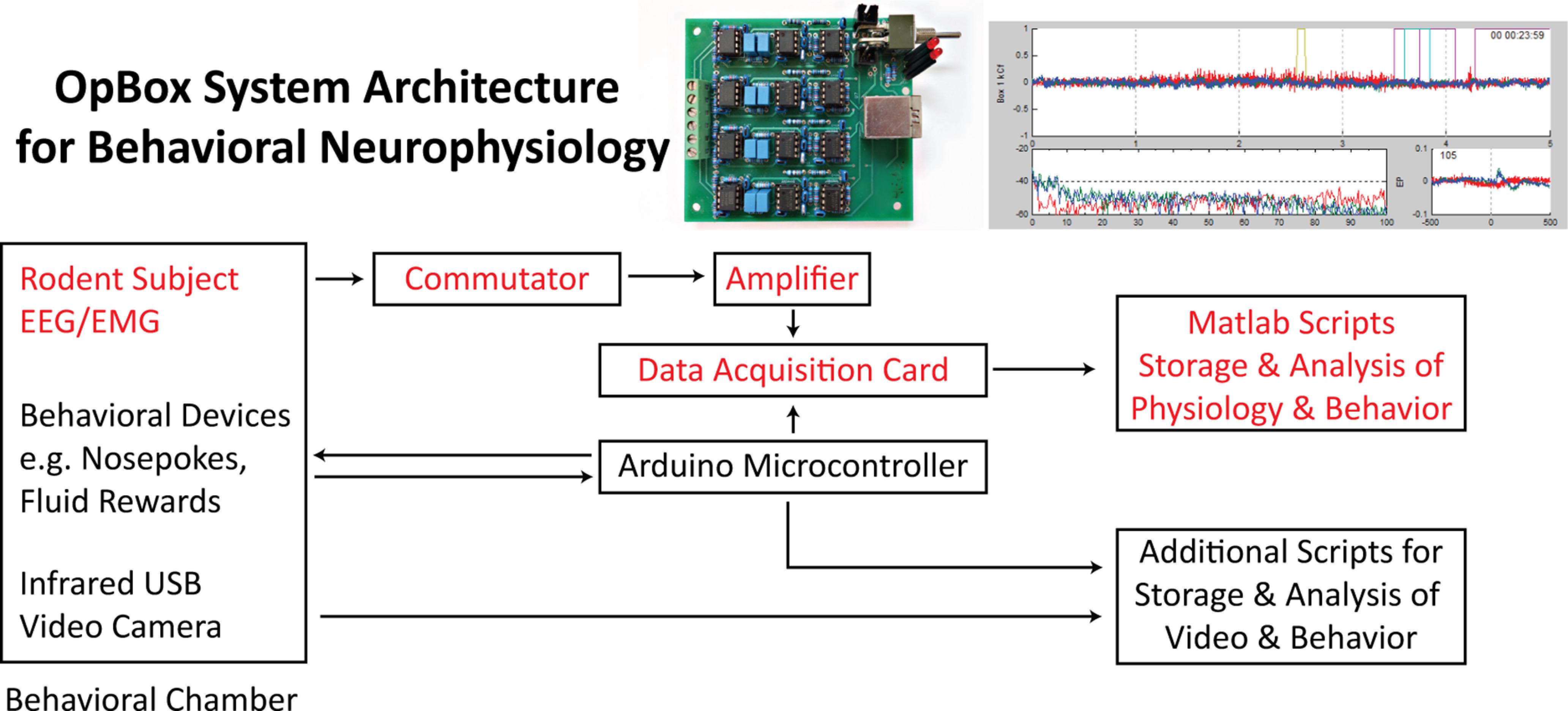

## Significance Statement

Current commercial solutions for electrophysiology recordings in awake, behaving animals can be relatively expensive, inflexible, or challenging to interface with custom-built equipment. Here, we describe the development of OpBox, an open source set of hardware and software to perform simultaneous acquisition of electroencephalography (EEG) and electromyography (EMG) signals from multiple behaving subjects. Together with off-the-shelf data acquisition systems, OpBox increases flexibility and lowers the cost for the simultaneous acquisition of electrophysiology from multiple behaving subjects.

## Introduction

The search for new understandings and treatments of neuropsychiatric disease depends in part on reproducible and translatable biomarkers (Drinkenburg et al., 2015; Jones et al., 2015). Electrical brain activity, as revealed by electroencephalography (EEG), can reveal brain states and dynamics (Berger, 1929) relevant to various clinical conditions (Engel and Romano, 1959; Tatum, 2001; Brown et al., 2010; Mashour and Hudetz, 2018; Chen and Koubeissi, 2019). Some of these conditions, such as delirium, can be clinically heterogeneous, making reproducible measurements of rigorous biomarkers particularly important (Trzepacz et al., 1992; van der Kooi et al., 2015; Kimchi et al., 2019). Reproducible *in vivo* electrophysiology experiments require the collection of data from multiple subjects, often for extended periods. While long-term recordings from multiple subjects can be made more efficient by performing them simultaneously, scaling up recordings requires careful coordination and consideration of costs and flexibility.

Open source devices can improve research reproducibility and flexibility, potentially at lower costs, including for electrophysiology experiments. By sharing not only the methodology of an experiment, but the equipment as well (such as through design files), variability caused by differences in tools can be minimized (White et al., 2019). Furthermore, open source communities allow for direct feedback on best practice and allow for more community input on development of new tools. Important steps in EEG studies in multiple subjects are amplification of low-power EEG signals and acquisition through coordinated digital systems. Several open source amplifier designs are available, including a simple, single-channel analog system (Land et al., 2001), and a more flexible, higher-channel count (≥16 channels) digital system (Wang et al., 2014; Frey, 2016; Siegle et al., 2017). Both of these systems were initially designed for recording from single experimental preparations, rather than from multiple subjects simultaneously. We have therefore identified design parameters more specifically suited for flexible, multisubject rodent EEG recordings, as would be desired for epilepsy, sleep, or delirium related translational studies (Trzepacz et al., 1992; Bragin et al., 1999; Oishi et al., 2016; Kadam et al., 2017), as well as other possible experiments. We identified goals of recording up to four channels per subject, at moderate sampling rates (500 Hz to 2 kHz), with filters appropriate for EEG recordings relevant to epilepsy and sleep (0.3–200 Hz). Most importantly, we desired to record from at least 10 subjects simultaneously with a target goal of less than $500 per simultaneous subject.

**Figure 1.**
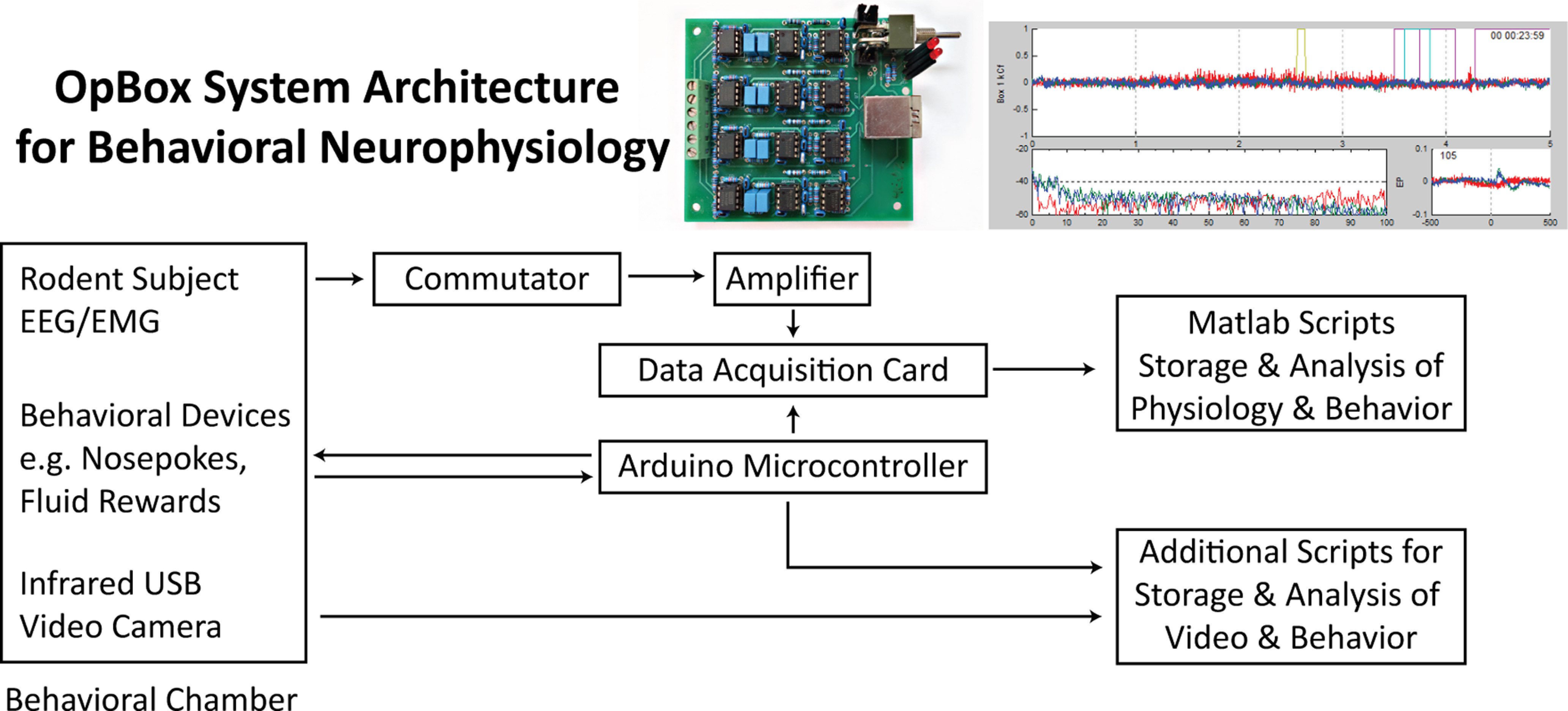
Overall architecture of the OpBox system. On the leftmost are the subjects and devices that may be placed within a behavioral chamber. Arrows in the top part of the figure indicate the flow of electrophysiology (EEG and EMG) data to the amplifier, data acquisition card, and software scripts. Additional arrows indicate the ability to add behavioral and video modules as well. Pictures of modules described in this paper in detail (OpBox amplifier and MATLAB GUI) are shown above their respective nodes.

We have developed an open-source system for simultaneous EEG/electromyography (EMG) acquisition from multiple rodent subjects, following the above design objectives, named OpBox. OpBox is a modular and flexible hardware and software system that constrains costs through judicious coordination of custom, open source circuits and software with off-the-shelf components and software commonly found in neuroscience laboratories (Fig. 1). Here, we verify our electrophysiology recording system by benchmarking it against commercially available amplifiers. We also demonstrate acquisition and visualization of EEG and EMG data from 12 subjects simultaneously, including real-time spectral analysis and averaged event-related potentials (ERPs) from subjects performing behavioral tasks.

## Materials and Methods

### Amplifier design

**Figure 2.**
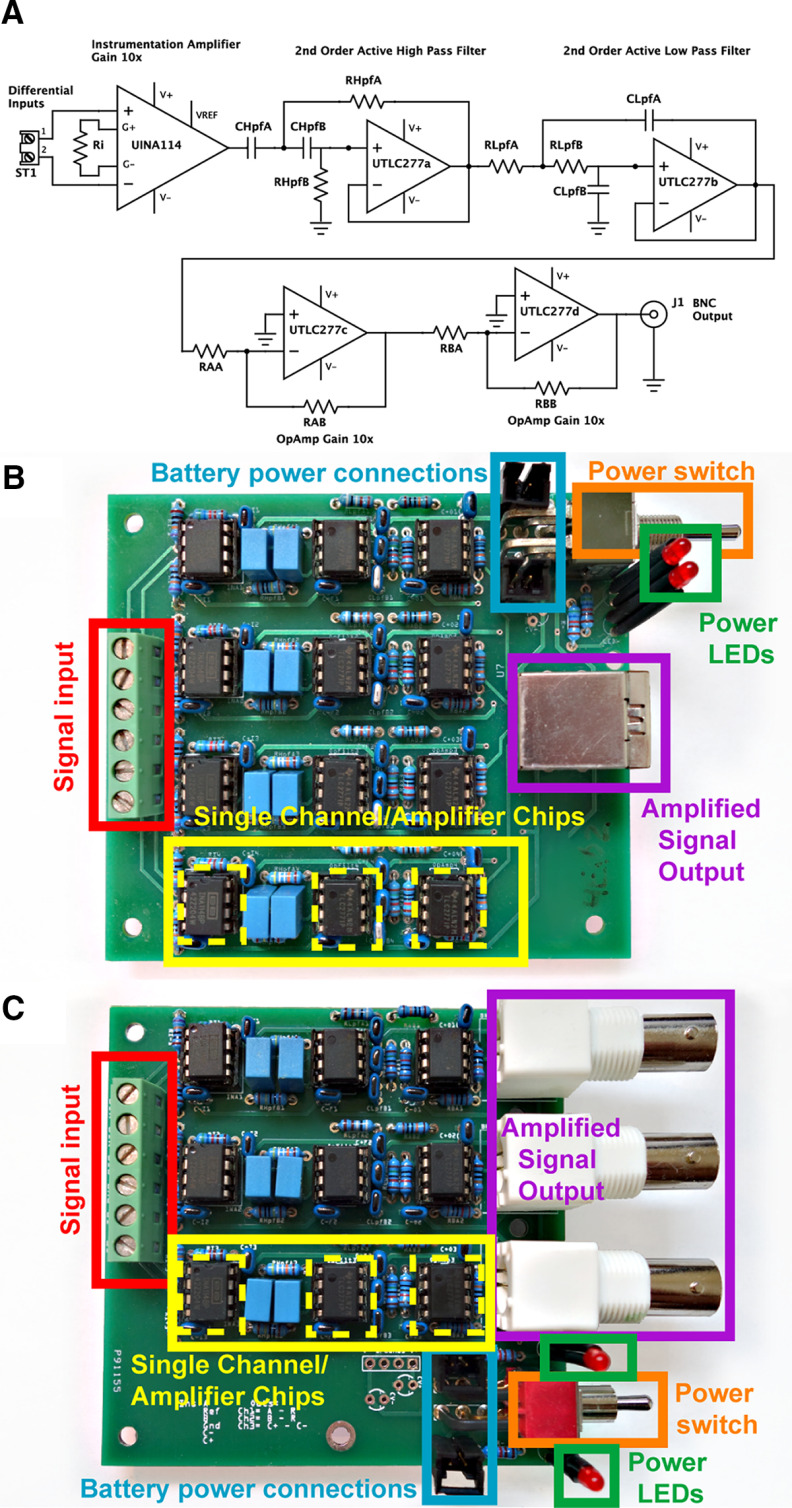
OpBox amplifier designs. ***A***, Circuit schematic for one channel of the electrophysiology amplifier. For clarity, power connections and decoupling capacitors are not shown. ***B***, Photograph of a four-channel amplifier PCB with all components. All channels in this four-channel amplifier share the same reference and there is an additional ground connection. ***C***, Photograph of a three-channel amplifier PCB with all components. In this three-channel amplifier, channels 1 and 2 share a reference, while channel 3 is a separate bipolar channel. Details for ***B***, ***C*** are as follows: Signal inputs via screw terminals for unamplified electrophysiology input signals (EEG and EMG). Single channel amplifier components. Amplifier chips are highlighted with dashed yellow outlines. Leftmost is an instrumentation amplifier (INA114, Texas Instruments) with low offset voltage (50 μV), drift (0.25 μV/°C), and high common-mode rejection (115 dB at G = 1000), set for a fixed voltage gain of 10×. The middle and rightmost chips are operational amplifiers (TLC277, Texas Instruments) with gain of 10× each, for a total system gain of 1000×. Each also implements one of two successive two pole active high-pass (0.28 Hz, Q ≈ 0.71) or low-pass (200 Hz, Q ≈ 0.71) filters in Sallen–Key configurations (6 dB/octave). Output connections for amplified signals. Four-channel amps use a single RJ45 (Ethernet) connector for all channels, three-channel amps use BNC connectors for each channel. LED indicators to check power connections and battery level for the positive and negative rails (±3V). Each power rail uses two AA batteries in series (+3 V and −3 V). Photographs in ***B***, ***C*** were adjusted to improve contrast and remove lab logo for blind review.

The main hardware component of our physiology system is a multichannel amplifier designed to collect electrophysiologic data, including EEG and EMG (Fig. 2). The OpBox amplifier is a four channel extension of a prior open source amplifier (Land et al., 2001). All four channels are referenced to a single electrode input, with an additional ground connection. Our design uses fixed filters, set by electrical component values, for a range of 0.3–200 Hz. Although we have not elected to use adjustable filters, the filter bandwidth could potentially be made adjustable by substituting a trim potentiometer for RHpfA (Fig. 2*A*) to adjust the high pass cutoff, and a dual potentiometer for RLpfA and RLpfB (Fig. 2*A*) to adjust the low pass cutoff. However, this might risk distorting the gain values of the amplifier. We cannot comment on the reliability of untested modifications of our design. The amplifier is powered by four AA batteries (total ±3 V) to reduce power line noise.

We connect electrodes from subjects to each amplifier using a short, passive cable via a commutator (details below within Multisubject simultaneous acquisition). Screw terminal inputs on the amplifier allow one to interface flexibly with various electrodes or cables, so labs can choose to use existing electrodes, cables, and commutator solutions from commercial companies, or integrate with other open source solutions (Medlej et al., 2019). Outputs from the four channel amplifiers are provided using an RJ45 or Ethernet jack, which allows one to transmit data via standard Ethernet cables to another RJ45 breakout connected to the data acquisition system. The cost of the four-channel amplifier, including printed circuit board (PCB) and components, is ∼$150 per amp when buying parts for 10 builds.

There are potential downsides of buffering the EEG signals on the acquisition side and not the animal’s head. Delayed buffering potentially increases the susceptibility of signal acquisition to environmental electrical noise. This may be problematic with long cables. We therefore mitigated this through early 1000× amplification at the top of the recording chamber and tested our system with typical cable lengths for rodent operant chambers, which allowed for longer cables (30’ BNC cables) before digitization by the data acquisition devices. The performance we report therefore may not be reflective of longer cables before amplification. Additionally, our system was designed for low impedance electrodes typically used for EEG studies, however, higher impedance electrodes for intracellular recordings may also be more vulnerable to delayed buffering. Because shielding may also mitigate the effects of delayed buffering, we provide details on the shielding we employed. Recordings were performed with both anesthetized and freely moving rats, as detailed below, to evaluate whether recordings remained satisfactory even when rats were moving, which may also be more susceptible to noise with delayed buffering.

In addition to the four-channel amplifier, we have designed a three-channel amplifier with similar components but a different referencing montage. Two channels share a common reference, but the third channel has a separate reference to enable a more specific bipolar configuration. We have found this configuration to be useful for separating EMG from EEG signals in studies involving sleep or convulsive seizures. Outputs for the three-channel amplifier are provided by dedicated BNC connectors for each channel. In practice, we have not routinely needed to use a line filter or further shielding with these amplifiers, although these features can be considered for use in electrically noisy environments.

We have used through-hole electronic components in the design of our PCBs, rather than surface-mounted components, to simplify bench fabrication of the amplifier more easily by lab members with limited electronics and soldering experience. While this design choice means the device is too large to be mounted on a rodent subject’s head, the multichannel, battery-powered amplifier is small enough to sit directly on top of a recording or behavioral chamber. We also elected to use an analog amplifier feeding into an off-the-shelf analog-to-digital data acquisition system capable of handling data from multiple amplifiers, to simplify implementation and reduce costs.

We designed our circuits within Fritzing (http://fritzing.org), an open source electronic circuit layout program that incorporates breadboard, schematic, and PCB views, and is commonly used in the Arduino community. We initially prototyped our circuits using breadboards, but then organized the design for a PCB. We exported the PCB as Gerber files for printing. These PCBs can be made in the lab using a desktop printer with etching solution or sent digitally to commercial manufacturers (e.g., $27.70 when purchased from Sunstone). In the Extended Data 1, alongside our PCB design files, we also provide bills of materials for our boards.

10.1523/ENEURO.0212-20.2020.ed1 Extended Data 1 PCB designs: amplifier PCB design files ready to be sent out for fabrication or modified to fit customized specifications. How to build + parts list: a document file containing detailed instructions for constructing the OpBox system, separated by component, with a spreadsheet of all parts used in the process of building the amplifier. Software scripts: MATLAB scripts necessary for running behavioral tasks and recording behavioral/physiological data. Includes example CSV spreadsheet files (InfoSubjects + InfoBoxes) and instructions for use. Download Extended Data 1, ZIP file.

### Physiology data acquisition

Our amplifiers produce analog outputs that are communicated to off-the-shelf analog-to-digital data acquisition devices. We initially explored using microcontrollers for analog-to-digital acquisition (DAQ); however, at the time of design, Arduino microcontroller ADCs were limited to 10 bits, and timing accuracy can be inaccurate by >0.1% (D’Ausilio, 2012), which can be more problematic for electrophysiology than behavior. Since that time, microcontrollers have become a more viable option, with commercially available boards such as the Teensy being employed to acquire 12-bit, 512-Hz EEG data (Blino.io). We elected to use National Instruments data acquisition devices given their widespread use in other otherwise open source electrophysiology systems, including often as benchmark devices for timing calibration (Rolston et al., 2010; Suter et al., 2010; Englitz et al., 2013; Campagnola et al., 2014). Potentially lower-cost alternative data acquisition devices such as LabJack are available but have yet to be tested with OpBox. We have successfully used OpBox with several National Instruments Data Acquisition devices (PCIe-6323, PCI-6225, USB-6009, USB-6210, and USB-6211). Amplified data are carried from the amplifier to the DAQ devices via twisted-pair (Ethernet) or coaxial (BNC) cables over an ∼3-m distance.

We have developed data acquisition scripts to manage data streaming from the amplifiers. National Instruments currently supports several programming environments, including MATLAB via the Data Acquisition Toolbox or C/Python. At the time of initial development, National Instruments provided official support for a MATLAB interface library, but did not provide official support for Python, so we followed other open-source acquisition efforts and developed our system in MATLAB (RRID:SCR_001622). We expect that a similar approach to that described below can also be used in Python. NI-DAQmx drivers should be downloaded from the NI website and installed before using OpBox.

A key design constraint was the ability to start and stop recordings from different subjects asynchronously. In most data acquisition devices, a single high-precision hardware clock starts and stops all channel acquisition simultaneously. Recording from multiple subjects might therefore require processing and saving data from all channels at once, with *post hoc* or retrospective splitting of individual subject data. In OpBox, we have instead coded an overarching set of scripts that allow for easy multisubject acquisition. OpBox supports the use of single or multiple hardware clocks across subjects. Even when recordings are acquired using a single hardware clock, the data streams from each subject are processed and saved independently by OpBox. End users simply identify subjects or amplifiers for which to start or stop recordings. Internally, all data from the DAQ system, including both used and unused channels, are consistently reported to the software scripts. However, our software uses two configurable text-based comma-separated value (CSV) spreadsheets to track: (1) which subjects are assigned to which recording boxes/amplifiers; and (2) which channels on our data acquisition devices are associated with those specific boxes/amplifiers. Assigning channels this way allows for flexibility in channel assignments for different amplifier configurations and makes it easy to track data selectively from only the active subjects at any given time, to process and save data from different subjects separately. This feature increases flexibility to stagger start and stop times and decreases further processing and storage requirements, allowing for data to be more easily displayed, processed, and saved in real time.

**Figure 3.**
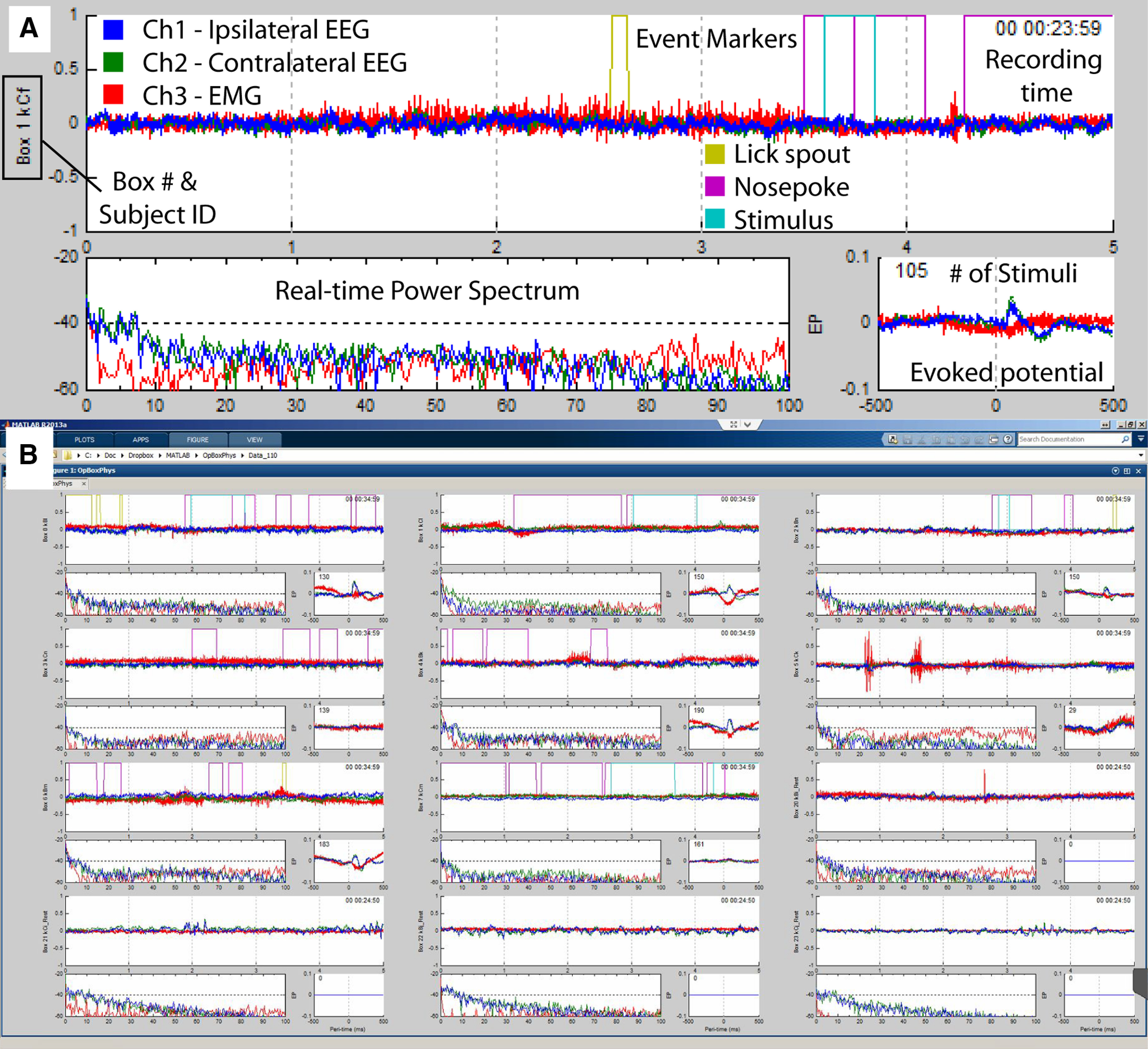
Simultaneous EEG/EMG recordings from rat subjects using OpBox scripts. ***A***, Data stream from a single subject. The largest plot shows overlaid amplified voltages from all channels (−1 to +1 V) over the past 5 s. The overlapping blue and green traces represent EEG, while the red trace is EMG from nuchal muscles. Rectangular traces in the EEG plot represent digital behavioral event markers (each color of yellow, magenta, and cyan represents a different type of behavioral event). Box number and subject ID are shown to the left of the plot. The top right of this plot shows elapsed time since the start of the recording. Below each EEG plot, a real-time FFT based power spectrum (0–100 Hz, plotted with log power on the *y*-axis) is shown to the left. The mean accumulated evoked potential (1-s total window length, auditory stimulus onsets are at time = 0 ms) is shown in the bottom right; *y*-axis is in amplified volts and the number of stimuli is indicated in the top left corner. This GUI is running in MATLAB on a Windows desktop computer. ***B***, Full GUI window showing simultaneous acquisition from twelve subjects. Eight rats are performing an auditory Go/No-go discrimination, each with their own mean evoked potentials displayed. The last four subjects are recordings of spontaneous behavior, i.e., while not performing a task, and thus have no evoked potentials. This multisubject GUI allows the experimenter to monitor all subjects simultaneously with a consistent configuration.

Real-time data processing includes online Fourier transforms to display frequency content of the electrophysiological data and evoked potential monitoring. These data are updated according to the internal data communication rate set by the MATLAB Data Acquisition Toolbox, for which we use the default of 10 Hz (100 ms). A screenshot and detail of a single subject’s stream during multisubject acquisition can be seen in Figure 3. Data are saved in a simple binary format for offline analysis, which can include analog physiology data, digital behavioral data or event markers, and rotary encoder data, for example, from linear treadmills in head-fixed preparations.

Using our software design, a single data acquisition device can be shared by multiple amplifiers. Therefore, a DAQ device with many channels (for example, 80 channels in the PCI-6225) can support many subjects (for example, 20 subjects with four channels each). Under this architecture, the primary scaling limitation of the number of channels that can be recorded from per computer is the number of PCI/e slots or USB connections for data acquisition devices collecting electrophysiology and behavioral data through analog and digital input channels. Expanding the system is primarily a matter of building new amplifiers to make use of all available input channels. PCI/e-based National Instruments devices can also be synchronized at the hardware level using a Real-Time System Integration (RTSI) cable (e.g., uniting a PCIe-6323 and PCI-6225 for a total of 112 analog input channels and 72 digital input/output channels). Hardware clocks of different USB devices cannot easily be synchronized, however; therefore, when using USB devices, all channels from a given individual subject should be kept on the same data acquisition device.

### Acquisition of additional data streams

OpBox software scripts can be configured to acquire additional data streams. For example, behavioral markers can be sent to digital input channels using transistor-transistor logic (TTL) signals or can be multiplexed and transmitted to analog input channels on the data acquisition device. The onsets of these behavioral events, such as rodent nosepokes, stimulus deliveries, or reward deliveries, can be used to demarcate sections of electrographic data for averaging automated, real-time ERPs. This is reliant on a low-latency behavioral system capable of sending out these TTLs, with one channel dedicated to each event of interest or through use of a multiplexed analog signal. Collecting behavioral data simultaneously with analog physiology data eliminates the need for *post hoc* attempts to synchronize timestamps. OpBox can also be used to simultaneously sample rotary encoder data, for example, from treadmills, using NI data acquisition devices that support rotary encoder data (e.g., USB-6211).

Lastly, OpBox also has the capability to display and save video data using any standard webcam supported by the MATLAB Image Acquisition Toolbox. Webcam video streams are synchronized with OpBox physiology by software. While software synchronization sets limitations on synchronization precision, it potentially allows users to employ less expensive cameras and may be sufficient if subsecond synchronization is required, rather than millisecond precision. In order to synchronize, every time data are updated from the NI DAQ to MATLAB (at the rate determined by the NotifyWhenDataAvailableExceeds property of the DAQ object, default 10 Hz), we register the time reported by the DAQ that the data were collected and also poll the MATLAB video input object at that moment to determine the number of the current frame just recorded (typically at 30 Hz). These data are saved independently for each subject and can be used for off-line video and physiology alignment, timestamp interpolation, and analysis.

### Performance benchmarking

We tested our OpBox system on a Dell Optiplex 9020 with an Intel Core i7-4770 CPU running at 3.4 GHz, with 16 GB of RAM and a 500-GB SCSI SSD (Samsung 840 EVO), running 64-bit Windows 7 (system cost ∼$1200 at purchase) and MATLAB R2013a (win64). Our scripts were originally developed in MATLAB version 2013a but have been tested to work up to version 2019b. We benchmarked the OpBox amplifiers using a National Instruments DAQ (PCIe-6323). We compared at least two different OpBox amplifiers to two systems previously available for purchase: (1) a Grass P55 AC preamplifier set to 1000× gain, with highpass/lowpass cutoff values set to 0.3 and 300 Hz, respectively, and line filter off; and (2) an open source system, based on the Intan RHD2000-series amplifier chips for signal amplification and digitization, similar to OpenEphys (Siegle et al., 2017). While the Grass amplifier is no longer commercially available for new purchase, a similar amplifier is available from AM Systems (Model 3000 AC/DC differential amplifier). For acquisition of the signal from the Intan system we used the RHD2000 USB interface board and the OpenEphys Graphical User Interface (GUI). To be most comparable to our recordings, we used highpass/lowpass cutoff values of 0.3 and 200 Hz, respectively. All systems were sampled at 1 kHz, except where indicated below.

To measure the noise floor for each amp, 5-min recordings were taken with each amplifier on, and a connection made between the differential inputs with a wire, a 1-kΩ resistor, and a 10-kΩ resistor. Another recording with all inputs unconnected was made as well. To better simulate noise that may be picked up by a tethered animal subject, we connected two channels of a shielded cable via a 10-kΩ resistor inside the behavioral chamber and the other ends to the differential inputs on the amplifier. The shield for the wire was connected to ground. Root mean square (RMS) values of the amplifier outputs were calculated for 10 non-overlapping 30-s segments. Mean RMS and SEM of windows were calculated for each combination of test and amplifier type. One-way ANOVAs were performed for each test condition to test for an effect from amplifier type on RMS values.

To measure the common mode rejection ratio (CMRR) for each amplifier, a 1-V peak-to-peak 60-Hz sine wave was output from an Agilent 33250A function generator and the positive lead was split and connected to both differential inputs. The negative lead was left floating; 5-min segments of recordings were taken from each amplifier type. The ratio of RMS of the output signal to the RMS of the input signal (from an ideal signal generated using MATLAB) was calculated for 10 non-overlapping 30-s segments for each amplifier type to determine the mean and SEM of signal reduction in decibels.

As a test of amplifying a known signal, a 100-μV peak-to-peak, 10-Hz sine wave signal was delivered to each amplifier input using the function generator. As this was below the output voltage range of the signal generator, a 100-mV peak-to-peak signal was stepped down by a factor of 1/1000 using a precision voltage divider circuit. We took 5-min recordings and compared amplifier outputs by computing the power spectrum densities of these recordings. To calculate signal-to-noise ratio (SNR) for each amplifier in these test recordings, we used the MATLAB SNR function (snr) to calculate the SNR in 20 non-overlapping 15-s windows for each recording. Mean and SDs of SNR values were taken for each amplifier and compared using a one-way ANOVA, with *post hoc* tests Bonferroni corrected for multiple comparisons.

In order to test the performance of inexpensive commutators, we performed an additional 30-s test signal recording in which the output of the voltage divider was sent to the amplifier input via our commutator. The commutator was spun by hand at ∼0.2 Hz using a stopwatch as a guide. Since this benchmarking recording setup does not allow us to spin our cables indefinitely, we spun clockwise for two revolutions, counterclockwise two revolutions, and then clockwise for two revolutions again. The raw signal was plotted alongside the approximate rotational position as well as a spectrogram of the recording made using 2-s windows and 80% overlap.

Cross talk between different channels of the same OpBox amplifier and between different amplifiers on the same DAQ was measured by recording unused channels during test signal amplification. Again, by using our function generator and voltage divider circuit, a 1-mV peak-to-peak, 10-Hz triangle wave was sent to the first channel of an OpBox amplifier. We recorded from the channel receiving the input, another unused channel on the same amplifier, and an unused channel from a second amplifier connected to an adjacent DAQ input, all for 5 min. RMS values of all three channels were calculated, as well as the RMS value of an idealized output (a 5-min, 500-mV, 10-Hz triangle wave generated in MATLAB) for 10 non-overlapping 30-s segments. Mean and SEM of RMS values for each channel were calculated and compared with the idealized signal.

For tests measuring phase distortion, gain, step response, and impulse response, we increased the sample rate in our OpBox MATLAB script from 1 to 100 kHz. For step and impulse response tests using the Intan devices, the max sampling rate of 30 kHz was used; however, since no means of bypassing the amplifier was possible for the Intan device, it was not included in phase distortion and gain analyses. To measure phase distortion, a 1-V peak-to-peak signal was split and sent both directly to our DAQ and to an input on the amplifier via voltage divider. Tests were performed with sine waves at frequencies of 5, 10, 25, 50, and 100 Hz. The 10-s recordings were Hilbert transformed and phase discrepancy was calculated for each frequency as the instantaneous difference in phase at each sample. Mean and SEM of phase difference was reported for each amplifier at each frequency tested. Using the same recordings, we calculated effective gain at each frequency by calculating the ratio of RMS of amplifier outputs to RMS of the input signals (simulated by dividing the direct-to-DAQ signal by 1000).

For step signals, a 0.05-Hz 1-mV unipolar rectangular wave was sent to the amplifier input using the function generator and voltage divider. Rise time was calculated as the time difference between an amplifier output reaching 10% and 90% of the max amplitude after delivery of signal. Plots were included to show amplifier response to step onset until outputs had settled completely. For an impulse signal, we used the signal generator and voltage divider to send a 500-μV unipolar, 1-ms pulse to one channel of each amplifier. Plots of a single impulse response were visually compared between amplifier types.

Measurements of input impedance for each amplifier were calculated by sending sine waves from the function generator and voltage divider at frequencies of 1, 5, 10, 25, and 50 Hz. This was done using a fixed gain, tuned so that the input was as large as possible without saturating the amplifier. At each frequency tested, recordings were made with the upper arm of the voltage divider output passed to the amplifier via a wire, and again passed via a 10-MΩ resistor. Recordings of each type were split into 20 non-overlapping windows, for which RMS was calculated. Input impedance values were then calculated using the formula: Input Impedance = 10 MΩ × RMS_10 MΩ_/(RMS_0Ω_ – RMS_10MΩ_) and are presented as medians and interquartile ranges.

To benchmark the synchronization of analog data and video streams, we flashed an infrared LED (Everlight IR333C/H0/L10) at both a webcam (Digital Innovations 4310100) and a phototransistor (TT Electronics/Optek Technology OP535B). Voltage across the phototransistor was measured by an NI DAQ (USB-6210). Data from both the webcam and the NI DAQ were recorded by OpBox within MATLAB R2018b using a Windows 10 desktop computer, and data streams were synchronized in software. The LED was flashed using an Arduino Mega, and was on for 1 s, and off for 2 s. We recorded 36 min of data. Offline, we identified LED onset times by thresholding each data stream at 1.5 times the median value. The difference between LED onset times in the two streams were compared, and descriptive statistics are reported in Results.

### Animal care and use

We also tested the performance of our amplifiers for recording EEG and EMG in animal subjects. All animal procedures were performed in accordance with the Massachusetts General Hospital animal care committee’s regulations. Young (three-month) and aged (24-month) male Brown Norway (*Rattus norvegicus*) rats were acquired from the National Institute on Aging Aged Rodent Colonies (RRID:SCR_007317) and housed in standard caging on a 12/12 h light/dark cycle with *ad libitum* access to food and water until the start of behavioral training, for which food restriction was used as a motivator for obtaining a caloric fluid reward from correct task performance. All experiments were started at ∼Zeitgeber time (ZT)3. Behavioral tasks consisted of a Go/No-go auditory discrimination, where responses to Go tones (pure tones at 3.5 or 12 kHz, ∼55 dB, 1-s duration) yielded 100 μl of reward delivered via a spout (Ensure clear), and responses to No-go tones (counter-balanced 12 or 3.5 kHz), were unpunished. Behavioral protocols were run locally on an Arduino Mega, a microcontroller and embedded computer system dedicated to each behavioral chambers. Behavioral results are not described in this manuscript, which is focused on the physiology system, but further details are available on request and hardware and software are freely available on our website (http://www.kimchilab.org/opbox/). Animal behavior was monitored using an infrared webcam (Digital Innovations P/N: 4310100) and training sessions typically lasted 1–2 h, with training typically taking at least two weeks before implantation.

After training, rats were implanted with four extradural skull screw electrodes and 2 EMG pads (Plastics One) under general anesthesia [ketamine 100 mg/kg (for induction and supplements if needed) + diazepam 10 mg/kg (for induction only)] and local anesthetics (lidocaine). Opioid pain management (buprenorphine 0.05 mg/kg) and NSAID analgesia (ketoprofen 4 mg/kg) were administered peri-operatively. Relative to bregma, extradural skull screw electrodes were located: 2 mm anterior and ±2 mm lateral from sagittal midline (bilateral); 3 mm posterior and ±3 mm lateral from sagittal midline (bilateral). The anterior right electrode was used as ground. The anterior left electrode was used as a reference to record two channels: an ipsilateral recording and a contralateral recording from the left and right posterior screws, respectively. EMG pads were placed in between nuchal muscles after blunt dissection posterior to the occiput. A bipolar EMG recording was made using the third channel, taken as the differential between the two pads. The screws, leads, and exposed skull were covered with dental cement (AM Systems). Daily ketoprofen administration continued for 2 d following the procedure. To avoid damage from cagemates chewing on headstage connectors, animals were housed individually after surgery. Rats were allowed to recover for at least one week. After recovery, behavioral testing was resumed, with concurrent electrophysiology data acquisition.

### Multisubject simultaneous acquisition

To demonstrate the capabilities of our system’s simultaneous, multisubject acquisition, we recorded EEG and EMG from 12 subjects simultaneously on a single computer. Subjects were placed in a custom-made acrylic box (Dan-Kar Plastics) inside of a laminated wooden box lined with acoustic foam (McMaster). Headstage connectors were attached using short cables (PlasticsOne P/N: 363–000 W/SPRING) to a commutator (Adafruit P/N: 736) at the ceiling of the chamber, which was connected to our three-channel OpBox amplifier. Amplifiers were connected via BNC cables to National Instruments breakout boards (NI P/N: 777145–02), which were connected to two data acquisition cards (NI PCIe-6323 and PCI-6225). A single instance of MATLAB on our desktop computer running Windows 7, installed with the NI-DAQmx drivers, handled data acquisition and visualization of incoming data streams, including raw EEG/EMG traces and real-time FFT power spectrum plots.

Physiologic data were synchronized with behavioral data streams at the hardware level by sending digital event markers from the Arduinos to the DAQ. Therefore, behavioral (or other digitally-encoded data) and physiological data could be supervised by different devices to maximize the number of ports available for each device type. Recording sessions lasted from 1 to 6 h depending on the experimental need. We united the data for further analysis offline though network and cloud services.

### Other hardware devices

In the Extended Data 1, we provide more details on the few other hardware devices in our system. These remaining components, like many others in OpBox, are chosen flexibly in modular fashion. For instance, we have tested our system with two different sets of EEG electrodes (PlasticsOne and custom-made screw electrodes) with similar results. For situations in which additional shielding is desired, we have employed prebuilt aluminum enclosures for our amplifiers with minor modifications to ground them and expose connections. We also have 3D-printed enclosures for our National Instrument interfaces to protect them and break out channels of interest in a patchbay of Ethernet or BNC connectors.

### Costs

The initial cost of OpBox is for 10 simultaneous subjects is ∼$5000 ($497 per subject) when buying parts for the initial 10 setups, including a desktop computer, MATLAB licenses, and National Instruments devices. A parts list spreadsheet with links to parts and cost is available as Extended Data 1. Commercial solutions, such as those offered by Pinnacle Technology and Biopac Systems can cost upwards of $35,000 or $42,000, respectively, for the initial 10 setups. Other open-source solutions like OpenEphys could be used, although not expressly designed for high subject, low channel count experiments and thus would still be more expensive (∼$14,000 for just the headstages and USB interface boards for an initial 10 simultaneous subjects).

### Code accessibility

The code/software described in the paper is freely available online at https://github.com/KimchiLab/OpBoxPhys. The code is available as Extended Data 1.

## Results

### Amplifier benchmarks

We benchmarked and compared our amplifiers using a variety of standard conditions. Noise testing experiments are reported in Table 1 as the average and SEM of RMS values with different resistance loads. OpBox amplifiers had comparatively low noise on all tests performed. When resistors were directly connected to the amplifiers in this particular setup, OpBox amplifiers had higher RMS than the Grass amplifier and lower than the Intan amplifier. We elected to use a consistent methodology for all amplifiers in the shielded cable tests; however, it should be noted that Intan headstage amplifiers are unlikely to have a cable run of this length, and thus this test does not necessarily reflect typical use for these amplifiers as compared with the Grass or OpBox amplifiers.

**Table 1:** Noise floor testing

Amplifier	Inputs shorted (μV rms)	Connected by 1 kΩ (μV rms)	Connected by 10 kΩ (μV rms)	Connected by 10 kΩ and 30-cm cable (μV rms)	Inputs open (μV rms)
OpBox A	1.322 ± 0.005	1.324 ± 0.009	1.337 ± 0.008	1.342 ± 0.006	1.708 ± 0.011
OpBox B	1.282 ± 0.006	1.284 ± 0.006	1.297 ± 0.006	1.306 ± 0.007	1.896 ± 0.021
Grass	0.554 ± 0.009	0.596 ± 0.046	0.589 ± 0.008	0.529 ± 0.018	12.756 ± 0.122
Intan	2.632 ± 0.009	2.708 ± 0.046	3.032 ± 0.055	22.107 ± 1.034	13.104 ± 0.508

Values from noise floor testing of the different amplifiers; 5-min segments of recordings were taken from each amplifier type with differential inputs shorted using a wire, connected using different resistors, or left completely unconnected. To mimic the effect of having EEG travel via a cable in a tethered subject recording, we also connected the amplifier inputs to a 10-kΩ resistor but at the end of a 30-cm shielded two conductor cable. The shielding of the cable was grounded. Mean RMS values of these 5-min segments were calculated using 10 non-overlapping 30-s windows. Mean and SEM values are reported for each combinations of amplifier type and testing criteria; *n *=* *10 non-overlapping 30-s windows for all table cells.

**Table 2:** Input impedance testing

Amplifier	1 Hz	5 Hz	10 Hz	25 Hz	50 Hz
OpBox	0.861 GΩ [0.859, 0.864]	0.866 GΩ [0.861, 0.868]	0.869 GΩ [0.866, 0.872]	0.885 GΩ [0.883, 0.889]	0.902 GΩ [0.899, 0.905]
Grass	0.100 GΩ [0.100, 0.101]	0.101 GΩ [0.101, 0.101]	0.102 GΩ [0.102, 0.103]	0.116 GΩ [0.116, 0.116]	0.204 GΩ [0.204, 0.205]
Intan	2.832 GΩ [1.926, 4.915]	2.301 GΩ [1.570, 2.861]	1.842 GΩ [1.775, 1.888]	1.391 GΩ [1.324, 1.471]	0.959 GΩ [0.901, 1.024]

Input impedance values for each amplifier at various EEG relevant frequencies. Two 3-min recordings were taken of sine wave signals, once using a 0-Ω upper arm resistor (wire) and once using a precision 10-MΩ resistor. Recordings were divided into 20 segments, and the RMS values were calculated for each segment. Ratios between RMS values using the different resistors were used to calculate the input impedances. Data are displayed as median [interquartile range].

We next tested CMRR to see how well OpBox amplifiers rejected common signals (1-V peak-to-peak, 60-Hz sine wave). CMRR tests show OpBox amplifiers have good performance rejecting common mode signals in this particular setup (*n *= 10: Grass = −61.54 ± 0.01 dB; OpBox A = −101.87 ± 1.49 dB; OpBox B = −111.69 ± 0.09 dB; Intan = −66.65 ± 1.10 dB). We note that the noise and CMRR values for the Intan recordings are higher than reported in their datasheet and further shielding would likely produce a result more comparable with their reported values; however, we kept conditions standard here between amplifiers as described.

**Figure 4.**
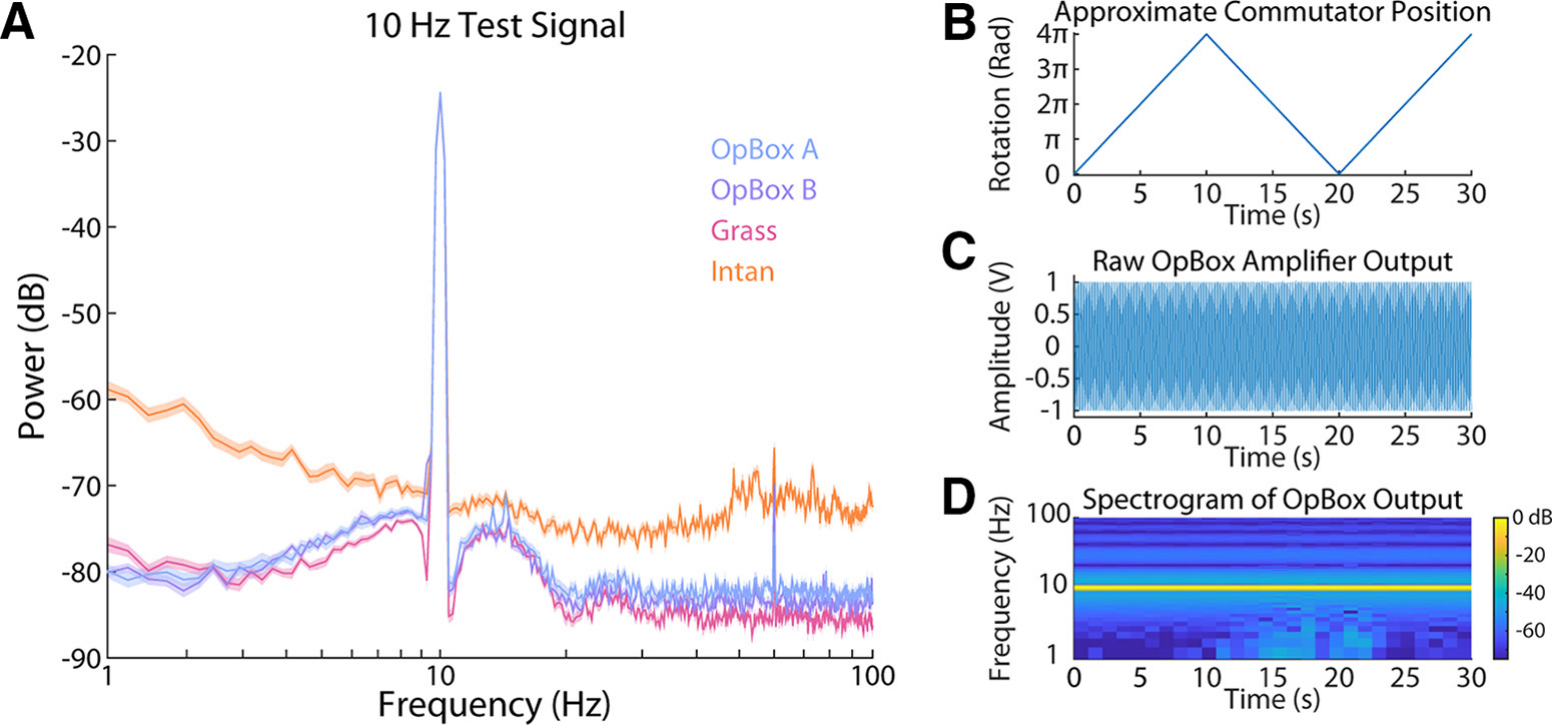
Amplification of a 10-Hz test signal. ***A***, Comparison of power spectrum density over a 5-min period, where a 10-Hz, 100-μV peak-to-peak sine wave was sent to one channel of each amplifier using a function generator and voltage divider circuit. Spectrograms were taken of the amplifier outputs using 4 s, non-overlapping windows (*n *=* *75 windows per amplifier). Spectra are shown with shaded 95% confidence intervals. No line filters were used. Signals were also sent via an inexpensive commutator used by our lab to verify their performance. Commutators were spun by hand. ***B***, Approximate rotational position in radians of our commutator where 0 is equivalent to the initial position. ***C***, Raw recording of the test signal. ***D***, Spectrogram of the signal.

OpBox amplifiers also reliably outputted clean, amplified signals from sine waves. As seen in Figure 4, power spectrums of test signal amplification show OpBox amplifiers were comparable to the SNR of other commercial amplifiers. Under these specific conditions, SNR values for amplifiers were as follows (mean ± SD): Grass = 26.72 ± 0.1732 dB; OpBoxA = 24.33 ± 5.54 dB, OpBoxB = 23.60 ± 4.11 dB, and Intan = 15.73 ± 2.38 (*p* < 0.01 for comparisons of Intan amplifier to each other amplifiers; *p* > 0.05 for comparisons between all other amplifiers). In cross talk tests, RMS analysis of the active channel (291.00 μVrms) showed a close value to an idealized triangle wave signal (288.79 μVrms). Unused channels had low RMS values both within the amplifier (1.84 μVrms) and between amplifiers (1.73 μVrms), suggesting minimal cross talk.

**Figure 5.**
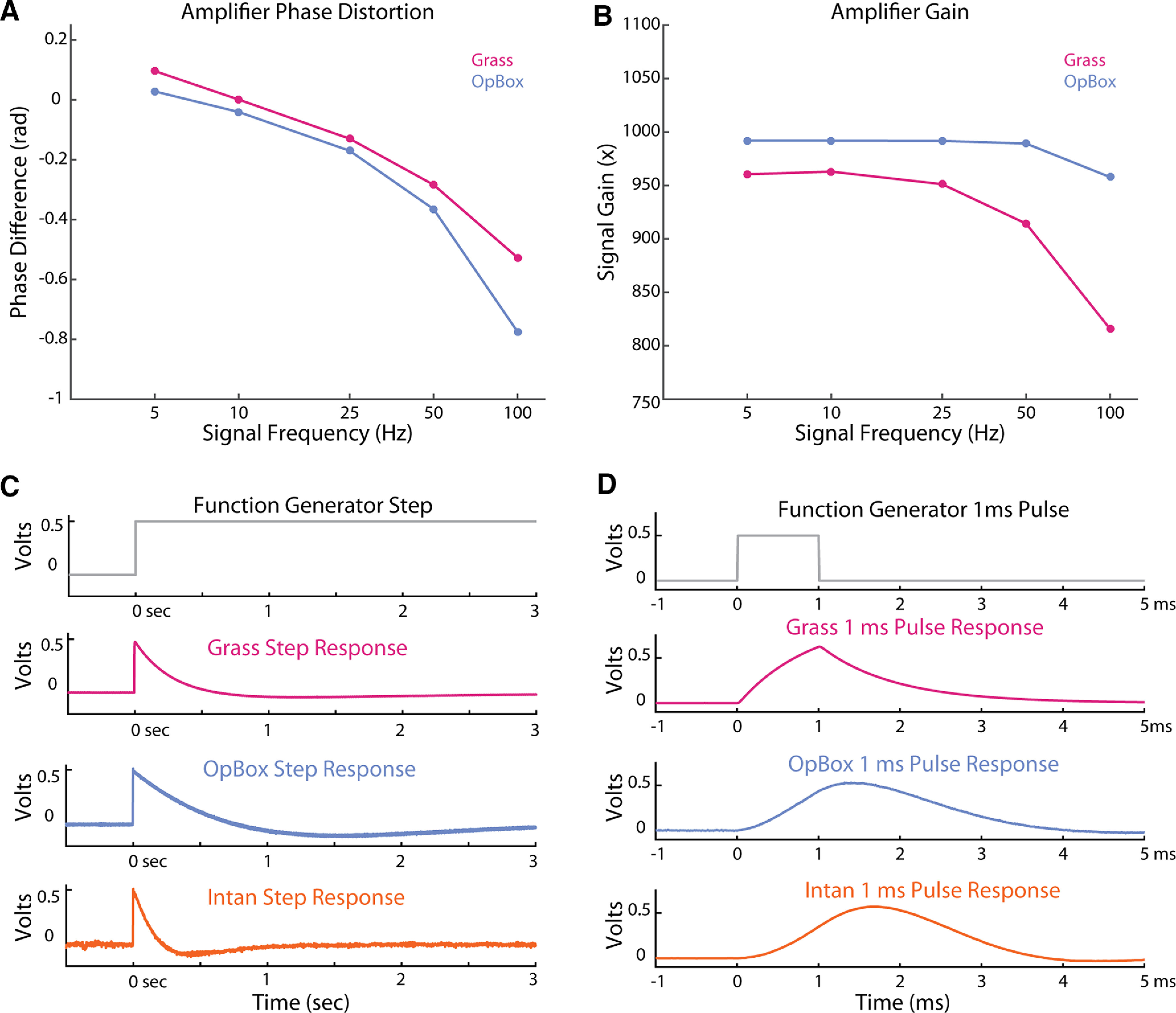
High sample rate tests. OpBox and Grass amplifiers were sampled at 100 kHz via OpBox scripts in MATLAB, and the Intan amplifier was sampled at their max rate of 30 kHz using the Open Ephys GUI. ***A***, Phase distortion from Grass and OpBox amplifiers at 5 different frequencies; 1-mV peak-to-peak sine wave signals at varying frequencies were split from a function generator and sent to one channel of the amplifier via a voltage divider as well as directly to the DAQ. Data from both inputs were sampled simultaneously at 100 kHz. Recordings were Hilbert transformed and the difference in phase angle was calculated for each frequency tested. ***B***, Measured gain for Grass and OpBox amplifiers at each frequency. Using the same recordings, RMS values over the entire 10 s were calculated and the ratio of amplified signals versus direct to DAQ signals divided by 1000 (to match the voltage divider output) are reported for each frequency. ***C***, Amplifier responses to a step response, a 10-s, 500-μV unipolar rectangular wave. A simulated version of the amplifier input is shown in the top plot. ***D***, Amplifier responses to an approximate impulse response, a 1-ms, 500-μV unipolar pulse. A simulated version of the amplifier input is shown in the top plot.

High sample rate tests, as seen in Figure 5, were performed to measure phase distortion, gain, step response, and pulse response. Analysis of phase distortion showed minor phase distortion effects from amplifiers that increase with frequency. Even at the highest frequency measured (100 Hz), distortion time remained steady (for *n *=* *10^6^ observations, standard error was ±0.001 rad for all Grass amp tests and ±0.005 rad for all OpBox amp tests) and never more than half a cycle behind the direct-to-DAQ recording for both Grass and OpBox amplifiers. OpBox amplifiers show steady gain with some roll-off at higher frequencies. For the step responses shown in the figure, rise times were 1.9 ms for the OpBox amp, 1.6 ms for the Grass amp, and 6.3 ms for the Intan amp. Impulse responses for each amplifier are included in Figure 5 as well.

Impedance tests showed good performance from OpBox amplifiers for all frequencies tested (Table 2). Grass amplifiers showed similar performance. The calculated values for the Intan amplifier were affected by increased variability, because of the large scaling factor in impedance calculations; i.e., variability of 1 GΩ in this test is resultant of <0.5% variability in ratios of recordings’ RMS values.

We benchmarked our synchronization protocol for analog data and video streams for a single camera by flashing an LED. LED onset times as captured by the video were delayed relative to onset times captured by a phototransistor going into the OpBox system by a mean of 66.1 ms (SD 13.2 ms). There was no significant drift between the first and last half of the 36-min recording (*t* test of delays from first vs second half of recording, *p* = 0.843).

### Animal data acquisition

**Figure 6.**
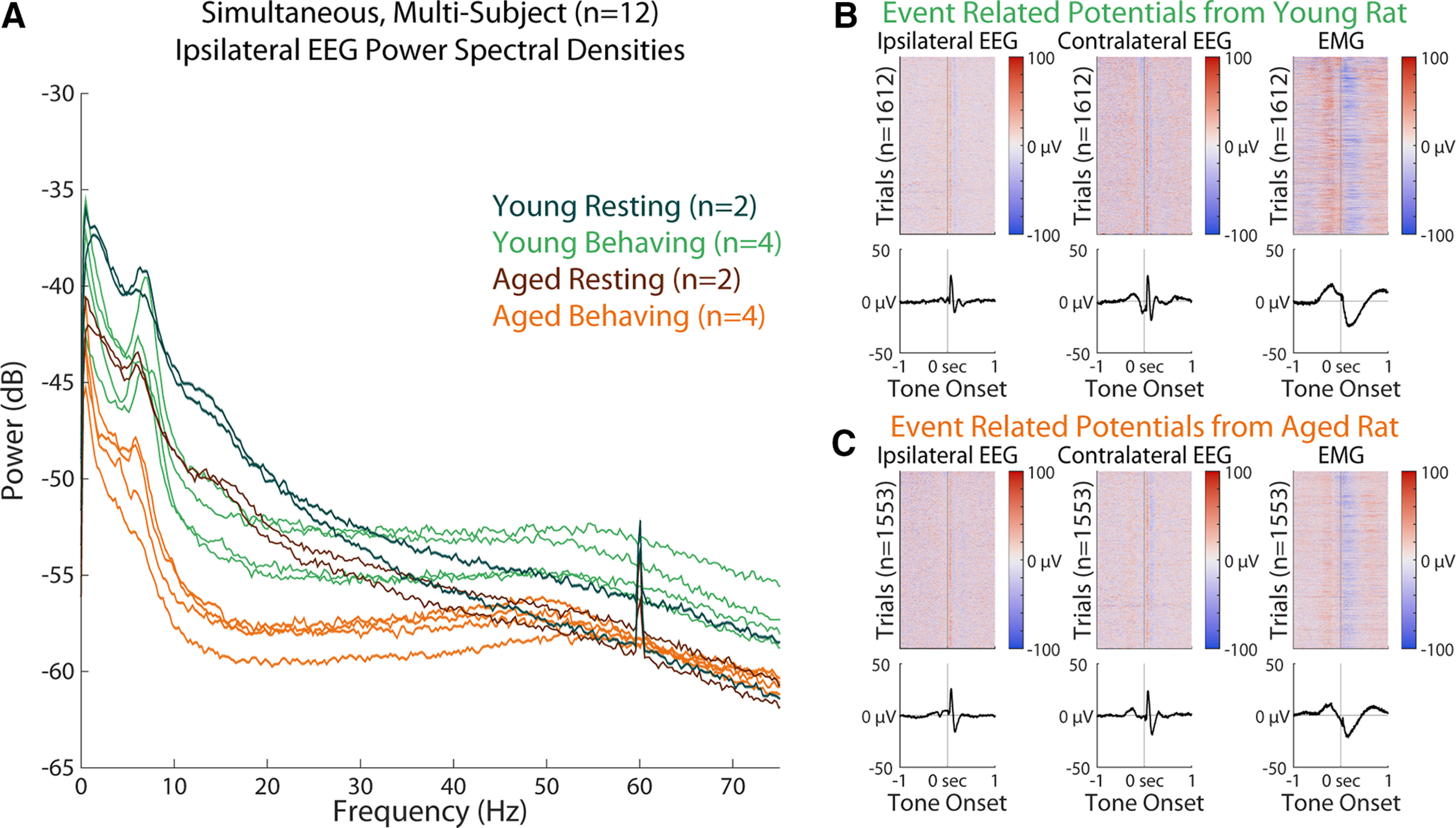
Multisubject session demonstrating the ability to record electrophysiology (EEG and EMG) data from 12 subjects simultaneously. ***A***, Combined plot of all 12 subjects’ ipsilateral EEG power spectrums. Each trace represents the mean power spectral density from one subject over 1 h of EEG recording. Traces are color coded by cohort, determined by age and whether subjects are performing a Go/No-go task or are resting. ***B***, ***C***, Evoked potential data from a young and aged subject, respectively, from the same session, demonstrating the consistent ability to synchronize EEG recordings with behavioral event triggers. Evoked responses shown were collected over the entire 4-h behavioral session. Each column represents a channel of EEG (ipsilateral or contralateral) or EMG. Top plots represent analog rasters for each trial, bottom plots represent the channel means, plotted with shaded SEM, although the high trial counts have reduced these intervals too much to visualize. Stimuli presentations were 1 s long and self-initiated by a nosepoke at least 100 ms in duration. Depending on task criteria, tones were 3.5, 6.5, or 12 kHz in frequency, at ∼55 dB. Time 0 s indicates tone onset, with preceding nosepoke registration at −0.1 s.

In Figure 6, we validated the simultaneous acquisition from 12 subjects. Recording from 12 subjects simultaneously (three analog channels, up to four digital channels per subject, each at 1 kHz) for over 6 h used 12% of total CPU power and 1.5-GB RAM. Battery life depended on the duration of use for each amplifier, but lasted more than a week when running continuously and more than a month when recording for 6 h/d.

We performed spectral analyses on the first hour of EEG recorded from each subject’s session and determined that all power spectrums showed physiologically relevant data in the form of band-specific power, which appeared to vary by subject age and behavioral state. We also analyzed ERPs from two subjects (one young and one aged) undergoing behavioral testing with delivery of tones, using digital triggers sent out by our behavioral system at the time of cue delivery, to validate the capability of our system to be sensitive to low-power events which are only discernible in time-locked averages.

## Discussion

We have developed OpBox, an open source system for acquiring electrophysiology data in behavioral experiments. We have successfully employed this system to record EEG and EMG in behaving rats, and have also used the system to record in behaving mice. Our system has been specially crafted to suit our experimental needs but is modular and flexible so that it can be tailored for different experiments. Flexibility is offered through dynamic channel assignment tailored to each subject specifically, the ability to acquire multiple signal types simultaneously, the ability to stagger start and stop times, and the resulting ability to combine subjects from separate protocols on a shared computer by way of these features. The scalability and modular design of our system allows for incremental expansion without a significant additional burden on each subsequent setup. After setting up a single amplifier, a second amplifier can still use the same computer, acquisition card, protocols, and analyses. Since the capability of our software in handling multiple subjects is not a limiting factor, adding subjects in software takes a minor amount of user input. OpBox simplifies multisubject protocols through the ability to have independent subject start times, synchronized data streams, and modular combinations of behavioral devices and cameras. We achieve similar performance per channel compared with a commercial general-purpose single channel amplifier at a fraction of the cost per channel, although with the trade-off of fixed filters and gain. Importantly for smaller labs, the system costs approximately an order of magnitude less on a per-subject basis than less customized commercial systems, although those may have their own advantages.

By using open source designs, collaboration and replicability can be achieved more easily and with less of a financial burden. The primary closed source but off-the-shelf components of our system are MATLAB and National Instruments DAQs. MATLAB is already used widely in many neuroscience laboratories. National Instruments devices are flexible, have a multitude of channels, and use well-maintained drivers (Parker et al., 2013). We decided that ease of implementation in these two domains were worth the use of proprietary designs. However, open source alternatives exist, for example, Python, a programming language which is increasingly used in laboratories as an alternative to MATLAB, and OpenDAQ, an open source USB-based data acquisition device. Through careful consideration of the synchronization capabilities of our off-the-shelf components, we can use of all available channels of the data acquisition devices to maintain reliable timing, including across PCI devices.

We anticipate that different labs will have different needs, and most labs that explore OpBox will use a subset of the open source modules, the amplifier and MATLAB scripts, we have designed. The modular nature of our system allows for users to only adopt certain parts, either specific hardware or software components without a loss in their functionality. This design philosophy allows for other equipment or data streams to be integrated with the OpBox system, as opposed to systems in which inputs and outputs are more tightly restricted. Consistent with the way in which we have benefitted from open source resources, we have made all of our hardware designs freely available on our website (https://github.com/KimchiLab/OpBoxPhys) under a Creative Commons Attribution-ShareAlike 4.0 International License (http://CreativeCommons.org/licenses/by-sa/4.0/), and software freely available under an MIT license (https://opensource.org/licenses/MIT).

This system has been fully implemented in our lab for more than four years and in the lab of a collaborator for more than half of that time. Our three-channel and four-channel amplifiers have proven sufficient for the recording montages desired in different experiments such as the behavioral experiments described here, experiments concerning EEG under anesthesia, recordings of sleep, and chronic EEG recordings of epileptic animals. We hope this system will also assist others in a wide range of applications perhaps beyond those described here, and that open source tools may help address ongoing concerns of replication and translation in neuroscience (Gleeson et al., 2017; White et al., 2019).

## Synthesis

Reviewing Editor: Mark Laubach, American University Decisions are customarily a result of the Reviewing Editor and the peer reviewers coming together and discussing their recommendations until a consensus is reached. When revisions are invited, a fact-based synthesis statement explaining their decision and outlining what is needed to prepare a revision will be listed below. The following reviewer(s) agreed to reveal their identity: Timothy Allen, Jakob Voigts.

Dear authors,

Thank you for submitting your manuscript to eNeuro. It received positive reviews from two researchers. Please see the reviews below and revise your manuscript to address all points that were raised. Your revised manuscript will be reviewed by me to ensure that all issues were addressed adequately. Please include a point-by-point reply to the reviews and also include a file showing the changes that were made (track changes). Thank you.

Regards,

Mark

Reviewer 1

My overall assessment is that this is a good open source contribution to EEG/EMG research, and should be of benefit to labs that perform this type of work. The authors have carefully demonstrated the performance of OpBox relative to Grass Instruments and Intan setups. OpBox can be run in multiple subjects simultaneously with independent stop/start commands. The authors also nicely provided a GUI based in MATLAB that can perform real-time data visualization and some processing (i.e., evoke potentials). I provide the authors with some specific questions and comments below:

I suggest naming the commercial amplifiers in the abstract.

The authors should briefly add specifics to the results in the abstract when comparing OpBox to commercial amplifiers. While the statement on recording 12 subjects simultaneously is useful, so would information about the comparative quality of the recordings.

Likewise, briefly state the cost savings and flexibility in more concrete terms in the abstract.

Table 1 reports variance as SEM. Was the sample size 10 in all cells?

Can the authors comment on the large noise increase in the Intan (and to a lesser degree the Grass) amplifiers when connected to a shielded cable, a pattern not observed at all with OpBox?

It’s not clear how OpBox improves reproducibility directly. It seems instead to increase experimental throughput, which might indirectly aid reproducibility, but often also comes at the cost of systematic error. For example, the same noise in the recording environment would be near identical in simultaneous recordings and could lead to misinterpretations about signal between groups recording on different days or in different labs. Can the authors expand on why simultaneous recording leads to reproducible measurements? I agree the simultaneous recordings are more efficient.

What flexibility does the OpBox offer specifically? Is it the dynamic channel assignment and signal acquisition?

Would a setup for 12 subjects cost $500 or 12 x $500? How do these prices compare to similar capability commercial products? Currently these comments are vague. Does this price include all necessary components (e.g., the NI acquisition boards)?

Line 124: What does “...apparently independent...” mean?

Figure 3B is a bit overwhelming. Is it necessary?

Are the Arduino setups described?

Line 191, Define CMRR.

Line 301 to 303. Please report summary statistics for SNR here. Does OpBox outperform Intan?

Figure 5C would benefit from a smaller x-axis range.

The discussion about modularity is not clear given that only the amplifier is displayed. Can the author add to Figure 1 showing all the modules in their setup?

Reviewer 2

There are a few clarifications and additions to the manuscript that would make it more useful:

-In the comparison between systems, please add measurements of input impedance across relevant frequencies.

-The introduction stating that commodity microcontrollers (the arduino example is given here) are not capable of sufficiently high fidelity or rate data acquisition is misleading. There are multiple projects that provide multichannel audio streaming from microcontrollers such as the teensy family. Using the nidaq family of AD interfaces is of course a valid choice, but the current manuscript makes it sound like it was the only one, and an extra sentence discussing other options would be nice. This also includes other cheap ADs such as the labjack family, etc.

- Can you add a brief discussion on the possible downsides of buffering the EEG signals only on the acquisition system side and not on the animal’s head? This seems relevant for scientists evaluating whether the system can work with their rigs, regarding cable length, electrode impedance, shielding, animal movement etc.

- Related: Can you provide data on how well cheap commutators work on this context?

- The reported noise data for the intan headstage seems surprisingly high. Could this have been cause by a discrepancy in grounding and shielding?

- The authors rightfully point out that the system can be built at low cost. Could you provide a table with a complete cost breakdown for a few representative setups?

- Would it be possible to at least discuss adding trim pots to set the filter bandwidth?

-What provisions exist in the system for synchronizing with other data sources such as cameras? Is this done in hardware (if so, please indicate this in the schematic figure), or by software. If this is done in software, please provide more details and benchmarks for this synchronization protocol.

- Fig. 2: there seems to be enough space to label the components. Just putting ‘a’,‘b’,‘c’ labels requires the reader to jump back and forth between caption and figure.

## Author Response

Synthesis of Reviews:

Significance Statement Comments for Author (Required):

N/A

Comments on the Visual Abstract for Author (Required):

Please consider adapting Figure 1 as your visual abstract. These extra images help raise interest and readership in manuscripts in eNeuro.

We have adapted Figure 1 to be used as a visual abstract (Fig0_VisualAbstract). To be clear about what the paper covers, we have highlighted in red the components of the flow chart we detail explicitly in our manuscript.

Synthesis Statement for Author (Required):

Thank you for submitting your manuscript to eNeuro. It received positive reviews from two researchers. Please see the reviews below and revise your manuscript to address all points that were raised. Your revised manuscript will be reviewed by me to ensure that all issues were addressed adequately. Please include a point-by-point reply to the reviews and also include a file showing the changes that were made (track changes). Thank you.

Reviewer 1

My overall assessment is that this is a good open source contribution to EEG/EMG research, and should be of benefit to labs that perform this type of work. The authors have carefully demonstrated the performance of OpBox relative to Grass Instruments and Intan setups. OpBox can be run in multiple subjects simultaneously with independent stop/start commands. The authors also nicely provided a GUI based in MATLAB that can perform real-time data visualization and some processing (i.e., evoke potentials). I provide the authors with some specific questions and comments below:

I suggest naming the commercial amplifiers in the abstract.

We have added those names in the abstract (line 17).

The authors should briefly add specifics to the results in the abstract when comparing OpBox to commercial amplifiers. While the statement on recording 12 subjects simultaneously is useful, so would information about the comparative quality of the recordings.

We have added a sentence in the abstract to state our amplifier’s comparable performance in specific benchmarking tests (line 18).

Likewise, briefly state the cost savings and flexibility in more concrete terms in the abstract.

We have amended the abstract to explicitly state costs ($500 per subject, line 10). Also, we specifically indicate in the abstract which types of data streams (analog EEG/EMG, event triggers from a behavioral system, and rotary encoder data) can be flexibly integrated (line 13).

Table 1 reports variance as SEM. Was the sample size 10 in all cells?

That is correct. The recordings were all cropped to exactly 5 minutes, which were then analyzed in 10 non-overlapping 30 sec windows. We changed the wording in the caption for Table 1 to clarify this: “n = 10 non-overlapping 30 sec windows for all table cells”.

Can the authors comment on the large noise increase in the Intan (and to a lesser degree the Grass) amplifiers when connected to a shielded cable, a pattern not observed at all with OpBox?

This pattern was unexpected by us as well, and so we returned to lab and made additional recordings in order to be certain there was not a discrepancy in recording quality. More aggressive shielding reduced the noise moderately. These tests were conducted in the same location as our other shielded cable noise tests which did not require such measures to obtain the values we previously reported.

With this in mind, the results OpBox is at least as resistant to noise interference as the Intan system under our typical recording conditions. It should be noted the typical distance between the amplifier and electrodes in the Intan system is on the order of millimeters, and thus the 30cm shielded cable is unlikely to mimic practical use of those amplifiers in contrast to the other two analyzed. We have included this caveat in our Results under Amplifier Benchmarks (line 369). We have also updated our results tables with those new noise values.

It’s not clear how OpBox improves reproducibility directly. It seems instead to increase experimental throughput, which might indirectly aid reproducibility, but often also comes at the cost of systematic error. For example, the same noise in the recording environment would be near identical in simultaneous recordings and could lead to misinterpretations about signal between groups recording on different days or in different labs. Can the authors expand on why simultaneous recording leads to reproducible measurements? I agree the simultaneous recordings are more efficient.

We have updated our introduction to better clarify how we believe OpBox can improve reproducibility (line 44). As the reviewer notes, increased experimental throughput will indirectly aid reproducibility. However, we also further clarified that by developing an open source system, we allow for more reproducible research across laboratories. Labs can more easily directly replicate not only our experiments but also equipment, in part by lowering the financial investment required for multiple institutions to use the same equipment. As opposed to commercial, closed source designs, this is not only facilitated by releasing a lower cost device but also more importantly by releasing the actual design itself, which can be evaluated even prior to building any new device. We believe this point is consistent with eNeuro’s support of open source hardware generally.

What flexibility does the OpBox offer specifically? Is it the dynamic channel assignment and signal acquisition?

We have added information regarding OpBox flexibility to the Discussion (line 425): 1) Dynamic channel assignment and 2) acquisition of multiple signal types contribute to the flexibility of OpBox. Additionally, 3) staggering of start and stop times for rodents run during concurrent/overlapping time windows allows for flexibility since a computer already running a task can accommodate starting additional recordings. By embracing a modular design, it is easier to combine subjects from separate experimental protocols on a shared computer this way, as we have with epilepsy, sedation, and delirium studies.

Would a setup for 12 subjects cost $500 or 12 x $500? How do these prices compare to similar capability commercial products? Currently these comments are vague. Does this price include all necessary components (e.g., the NI acquisition boards)?

We have clarified the goal cost in the introduction from “$500 per recording setup” to “$500 per simultaneous subject” (line 59). We have added a “Costs” subsection to the methods that clarifies that all necessary components are included in the cost calculation with specific details. We also compare these prices to commercial products with similar capabilities in this subsection.

"The initial cost of OpBox is for 10 simultaneous subjects is approximately $5000 ($497 per subject) when buying parts for the initial 10 setups, including a desktop computer, MATLAB licenses, and National Instruments devices. A parts list spreadsheet with links to parts and cost is available as Extended Data. Commercial solutions, such as those offered by Pinnacle Technology and Biopac Systems can cost upwards of $35,000 or $42,000, respectively, for the initial 10 setups. Other open-source solutions like OpenEphys could be used, though not expressly designed for high subject, low channel count experiments and thus would still be more expensive (approximately $14,000 for just the headstages and USB interface boards for an initial 10 simultaneous subjects).”

Line 124: What does “...apparently independent...” mean?

We have changed this text for clarification, by removing the word apparently and being clearer about what aspects are actually independent. “OpBox supports the use of single or multiple hardware clocks across subjects. Even when recordings are acquired using a single hardware clock, the data streams from each subject are processed and saved independently by OpBox. End users simply identify subjects or amplifiers for which to start or stop recordings.” (line 150)

Figure 3B is a bit overwhelming. Is it necessary?

We appreciate the reviewer’s comment. Figure 3B is intended to show that our GUI has the capability to simultaneously display real time data from all subjects currently being recorded on a single monitor.

We have clarified in this caption that “This multi-subject GUI allows the experimenter to monitor all subjects simultaneously with a consistent configuration.” Seeing data streams from all subjects at once highlights an important feature of our system (simultaneous recordings) we believe others may appreciate as well.

Are the Arduino setups described?

While the Arduinos are important to our behavioral experiments, we decided the scope of this paper was better limited to just the electrophysiology components of the OpBox system. Our Arduino code and shield PCB designs are also available from our laboratory GitHub, which has now been included in the Animal Care and Use subsection of the Methods (line 306).

Line 191, Define CMRR.

We have defined CMRR in this instance (now line 228) and changed where it was defined later in the paper (line 372) to just CMRR.

Line 301 to 303. Please report summary statistics for SNR here. Does OpBox outperform Intan?

We have added SNR summary statistics to the Results (now line 381-4). Both OpBox and the Grass amplifiers, which are battery powered, appear to outperform Intan under these specific conditions.

Figure 5C would benefit from a smaller x-axis range.

We have updated the figure so both 5C and 5D have a smaller x-axis range.

The discussion about modularity is not clear given that only the amplifier is displayed. Can the author add to Figure 1 showing all the modules in their setup?

We have clarified our discussion about modularity to indicate that it is our design principle as opposed to other systems which required a single unified design (line 453). Users may be interested in substituting or eliminating any of the devices indicated in the various boxes, depending on their experimental needs. We have added pictures of our amplifier and MATLAB GUI to Figure 1 in order to illustrate the modules covered by this paper.

Reviewer 2

There are a few clarifications and additions to the manuscript that would make it more useful:

-In the comparison between systems, please add measurements of input impedance across relevant frequencies.

We have added Table 2: Input Impedance Testing in which we include impedance measurements for different amplifier types across relevant frequencies.

-The introduction stating that commodity microcontrollers (the arduino example is given here) are not capable of sufficiently high fidelity or rate data acquisition is misleading. There are multiple projects that provide multichannel audio streaming from microcontrollers such as the teensy family. Using the nidaq family of AD interfaces is of course a valid choice, but the current manuscript makes it sound like it was the only one, and an extra sentence discussing other options would be nice. This also includes other cheap ADs such as the labjack family, etc.

We appreciate the reviewer highlighting these other options and did not want to give the impression that the NI DAQ family was the only option. We have added discussion in the Physiology Data Acquisition subsection of the Methods highlighting other options including explicit mentions of Teensy and LabJack (lines 128 & 133).

- Can you add a brief discussion on the possible downsides of buffering the EEG signals only on the acquisition system side and not on the animal’s head? This seems relevant for scientists evaluating whether the system can work with their rigs, regarding cable length, electrode impedance, shielding, animal movement etc.

We have added a brief discussion on the downsides of delayed buffering in the Methods section under Amplifier Design (line 90). Here, the choice was made not to include a buffer amp because of the additional complexity required. The buffer either has to have a battery, meaning unacceptable weight and size, or additional wires to deliver power and a source for that power, meaning an increased design complexity of the main board and requirements for unused channels in a commutator which we wanted to avoid. It is not out of the question to add such a buffer, but we opted for simplicity with the current design.

- Related: Can you provide data on how well cheap commutators work on this context?

To show the performance of the inexpensive commutators, we made an additional test signal recording while spinning the commutator to demonstrate reliability of signal transduction through them. We have amended Figure 4 to include plots from this test.

- The reported noise data for the intan headstage seems surprisingly high. Could this have been cause by a discrepancy in grounding and shielding?

We were also surprised by the higher noise floor in these amplifiers, which perhaps relates in part to the use of an AC power supply for the Intan system rather than battery power as for the other amplifiers. The initial recordings were all made in the same location of our laboratory space, including a grounded Faraday cage which was also tied to the ground of our amplifiers. We since returned to lab to make additional recordings, using more-aggressive shielding around cables. This reduced the noise moderately, for which we have updated Table 1. Our new reported values for the Intan system continue to show that OpBox is at least as resistant to noise interference under the specified conditions.

- The authors rightfully point out that the system can be built at low cost. Could you provide a table with a complete cost breakdown for a few representative setups?

We have included a parts list spreadsheet as extended data, and mentioned so in the Methods, under the Cost subsection.

- Would it be possible to at least discuss adding trim pots to set the filter bandwidth?

We have included a discussion on adding trim pots to set the filter bandwidth in the Amplifier Design subsection of the Methods (line 75). Since we have not actually tested such a design, we have also included a caveat that these untested modifications may produce undesirable changes in amplifier performance.

-What provisions exist in the system for synchronizing with other data sources such as cameras? Is this done in hardware (if so, please indicate this in the schematic figure), or by software. If this is done in software, please provide more details and benchmarks for this synchronization protocol.

We have provided more details and benchmarks for camera synchronization. Information on how we synchronize the streams is now provided in the Acquisition of additional data streams subsection of the Methods (line 194). Information on how we benchmarked this protocol is now provided at the end of the Performance Benchmarking subsection of the Methods (line 284). Benchmark results are now provided at the end of the Amplifier Benchmarks subsection of the Results (line 402).

- Fig. 2: there seems to be enough space to label the components. Just putting ‘a’,‘b’,‘c’ labels requires the reader to jump back and forth between caption and figure.

We appreciate the reviewer’s suggestion and have included component labels on the figure.
