# Supplementary material for: OpBox: Open Source Tools for Simultaneous EEG and EMG Acquisition from Multiple Subjects
Source: eNeuro. 2020 Oct 27;7(5):ENEURO.0212-20.2020. doi: 10.1523/ENEURO.0212-20.2020 (PMC7598913; doi:10.1523/ENEURO.0212-20.2020)
Supplement: Extended Data 1 — PCB designs: amplifier PCB design files ready to be sent out for fabrication or modified to fit customized specifications. How to build + parts list: a document file containing detailed instructions for constructing the OpBox system, separated by component, with a spreadsheet of all parts used in the process of building the amplifier. Software scripts: MATLAB scripts necessary for running behavioral tasks and recording behavioral/physiological data. Includes example CSV spreadsheet files (InfoSubjects + InfoBoxes) and instructions for use. Download Extended Data 1, ZIP file. [file enu-eN-OTM-0212-20-s02.zip › How to Build and Parts List/HowTo_Amplifier.doc]

***OpBox EEG/EMG Amplifier***

Parts are listed in the OpBoxPartsList spreadsheet, under SystemPart: Physiology: Amplifier

Start with an amplifier printed circuit board.

The following components need to be soldered to the board, and we recommend doing so in the following order (lowest to tallest). There are numerous soldering tutorials that can be easily found online. In general, we hold through hole components in place by inserting them and bending the legs. Alternatively, we sometimes use tape to hold them to the board before soldering.

Resistors

| Precision | Value | Color | #/Ch | Place(s) | Notes |
| --- | --- | --- | --- | --- | --- |
| 0.1% | 100k |  | 2 | RI+, RI- |  |
| 1% | 5.62k |  | 1 | RI | *There are 2 additional 5.62k resistors for the whole board, from battery to LEDs* |
| 1% | 9.09k |  | 1 | RLpfA |  |
| 1% | 10k | Red | 2 | RAA, RBA |  |
| 1% | 15k |  | 1 | RLpfB |  |
| 1% | 100k | Orange | 2 | RAB, RBB |  |
| 1% | 499k |  | 1 | RHpfA |  |
| 1% | 1M |  | 1 | RHpfB |  |

Bend resistor legs away from other holes. Avoid cold solder joints, especially for ground connections, which have the tendency to dissipate heat away. Clip extra leg leads when done.

**Chipsets**

3 per channel, U hole/8 marking towards top of board. Bend legs inward.

Capacitors

| Precision | Value | Color | #/Ch | Place(s) |
| --- | --- | --- | --- | --- |
| 0.1% | 0.047u | Blue, black stripe -> mark as silver | 1 | CLpfB |
| 1% | 0.1u | Blue, black stripe | 7 | Rest of Capacitor locations |
| 1% | 1u | Blue box (orient parallel to chipsets) | 2 | CHpfA & CHpfB |

All of above capacitors are bidirectional

Clip when done

**Screw Terminal**

Make sure open gates face out

**Switch**

1 DPDT switch

**Socket for Battery terminals**

Mount with tab facing "down" towards bottom edge of board. Will crowd switch, but done this way on first amp and slightly easier to remove battery leads this way, which are asymmetric due to tab

**Outputs**

We have experimented with several different outputs. For instance, the 3 channel boards use BNC jacks as outputs, 1 per channel. The 4 channel boards use an Ethernet RJ45 jack. Please note that this should NOT be plugged into a computer.

**Red LEDs**

Long leg goes into the box marking

It may be helpful to leave the LED legs long, projecting it above the board, so that the LED can be more easily seen. In this case we cut 1/2" of 1/16" heat shrink tubing so that the LED sits above the height of the switch.

**Insert amp chips**

Once all soldering is done, insert the amplifier chips into the sockets

| Name | #/Ch | Place(s) |
| --- | --- | --- |
| INA114 | 1 | INA |
| TLC227 | 2 | OpFilt & OpAmp |

**Put 4 rubber bumper feet on bottom**

Make sure not to touch active electrical connections

**Make battery holders**

Crimp on 22-26G crimps to each wire

Insert Black wire into dot side, Red into other side. The small socket tabs should face the dot side of the connector, in order to catch in the holes.

**Test amplifier with Function Generator**

Test that the amplifier works as expected. Connect a function generator to the amplifier. The function generator output/signal should go to all of the positive differential signal inputs, and the ground should go to all of the negative differential inputs/references and the amplifier ground. We test with a 10 Hz, 1mV sine wave. Make sure that each channel gives an appropriate output (1000x gain = 1V) and that noise levels are equivalent across channels (<5dB difference). We use an Agilent 33250A function generator for testing.
