# Supplementary material for: OpBox: Open Source Tools for Simultaneous EEG and EMG Acquisition from Multiple Subjects
Source: eNeuro. 2020 Oct 27;7(5):ENEURO.0212-20.2020. doi: 10.1523/ENEURO.0212-20.2020 (PMC7598913; doi:10.1523/ENEURO.0212-20.2020)
Supplement: Extended Data 1 — PCB designs: amplifier PCB design files ready to be sent out for fabrication or modified to fit customized specifications. How to build + parts list: a document file containing detailed instructions for constructing the OpBox system, separated by component, with a spreadsheet of all parts used in the process of building the amplifier. Software scripts: MATLAB scripts necessary for running behavioral tasks and recording behavioral/physiological data. Includes example CSV spreadsheet files (InfoSubjects + InfoBoxes) and instructions for use. Download Extended Data 1, ZIP file. [file enu-eN-OTM-0212-20-s02.zip › How to Build and Parts List/HowTo_CableCommutatorConnections_Harwin3mm.doc]

**Physiology Connections**

***Commutator: How to modify commutator for 3mm Harwin connectors***

Parts are listed in the OpBoxPartsList spreadsheet, under SystemPart: Physiology: Commutator

**Cut back wires and strip**

Cut central 6 wire end of commutator to 2cm length (from base of wire to tip)

Strip new blunt ends back 3mm (using auto-strippers)

**Crimp on contacts and solder lightly**

Use male contacts for 3mm TE Connectivity pins

Solder male contact just where the wires are (basically quick tin for extra reliability after crimp)

**Insert connectors into housing**

Insert in following order (with knob down, wires on top).
From left to right on knob side (6, 5, 4): Black, White, Red
6 = Black
5 = White
4 = Red

From left to right on flat side (1, 2, 3): Green, Blue, Yellow
1 = Green
2 = Blue
3 = Yellow

Final 2x3 arrangement, looking from top/wire ends & front/knob side towards you/right:

| Yellow | Blue | Green |  | Green | Red |  |
| --- | --- | --- | --- | --- | --- | --- |
| Black | White | Red |  | Blue | White | Knob |
|  | Knob |  |  | Yellow | Black |  |

Make sure contacts are parallel to housing (but otherwise ok up or down). Use tweezers/forceps to insert (push &/or pull from other side)

**Mark and test connector fit**

Put silver marking on sides of tab

Check with a female contact connector to make sure pins are aligned and do not slip back

***12 Channel Commutator***

Follow the steps above for the 6 Channel commutator but for all 12 channels. The final position/orientation for colors in the Harwin connector are shown below

| 7 | 8 | 9 | 10 | 11 | 12 |
| --- | --- | --- | --- | --- | --- |
| Red | White | Black | Gray | Brown | Light Blue |
| Green | Blue | Yellow | Orange | Purple | Gold/Tan |
| 1 | 2 | 3 | 4 | 5 | 6 |

Looking at Male end of commutator, look at numbers on non-tabbed housing

***Cable: How to modify Plastics1 free wire ended cable for use with a Harwin 3mm Connector (Female sockets)***

Parts are listed in the OpBoxPartsLIst spreadsheet, under SystemPart: Physiology: Cable

**Prepare cable and cut to desired length**

Cut a 3cm piece of 3/16” heat shrink tubing and slip it down over the wires & spring

Cut back whole cable including spring to length of 34-36cm, flush cut with heavy shears/tin snips (to get through internal rope too)

**Separate individual channels and strip wires**

Using needle nose pliers to hold the inner wires & plastic insulation, pull spring back 5cm and tape to hold back temporarily

Strip 2cm of inner plastic insulation using a scalpel while rolling the cable back and forth underneath it

Use auto strippers to pull off 3mm of insulation. Tightness setting should be 3 back from max/+ (0,1,2, 3). Mostly nicks insulation, does not fully strip it or cut wires, so pull off very gently, should be 3 wires & ropes left

**Apply crimp contacts and fit into housing**

Load the crimp contact in the crimper so that only the insulation grip & wire grip are in the crimper (only 2 end sets of contacts). The other set that looks like a grip are actually an orientation barrel/guides (keep just outside of crimper)

Crimp wires onto contact

Tin crimp joints with solder for extra stability

Insert contacts into housing according to Plastics1 color configuration. Make sure guides are oriented correctly (only 1 orientation of 4 will work: ENG_SS_114-13000_C.pdf):

Tab side: Left to Right: 6 Black, 5 White, 4 Red

Flat side: Left to Right: 1 Green (Arrow), 2 Blue, 3 Yellow. These insert "upside-down" relative to 4-6. Tabs are downwards/inside

On low setting, heat shrink bottom half of tubing away from connector

Put silver marking on sides of tab and label with a number on the back

Epoxy can be added where wires enter housing to protect exposed channels from rodent chewing
